# Hiatal Hernia to Bochdalek: A Case Report

**DOI:** 10.7759/cureus.43859

**Published:** 2023-08-21

**Authors:** Anjani H Turaga

**Affiliations:** 1 Medicine and Surgery, Gandhi Medical College, Hyderabad, IND

**Keywords:** bochdalek hernia, ards, misdiagnosis, adult bochdalek hernia, bochdalek, hiatal hernia

## Abstract

Hiatal hernias and Bochdalek hernias are two types of diaphragmatic hernias that present with similar symptoms. However, they differ in their etiology and anatomical location. In this case study, we present the clinical features and management of a patient who presented with symptoms suggestive of a hiatal hernia but was later diagnosed with a Bochdalek hernia. Our case has a 64-year-old female patient who presented with chronic obstructive pulmonary disease, hypertension, and gastroesophageal reflux disease. During her pulmonologist-ordered imaging, which included a CT scan, the report showed a large 8 cm hiatal hernia. Due to her condition, she was scheduled for a hiatal hernia repair, along with a transoral incisionless fundoplication (TIF) procedure. During the operation, a large defect was seen in the left hemidiaphragm with herniation of bowel loops into the chest cavity. It was confirmed to be a Bochdalek hernia. The surgeon proceeded to continue the laparoscopic repair, pulling the bowel back into the abdomen, and using the falciform ligament of the liver to buttress the diaphragm. The surgery was a success, and the patient had no postoperative complications. This case serves as a reminder that a high degree of suspicion is required for the diagnosis of Bochdalek hernias, especially in patients with atypical presentations or imaging findings suggestive of an alternative diagnosis, such as a hiatal hernia. The patient had chronic symptoms of various gastrointestinal and respiratory comorbidities, which should serve as a caution for clinicians to carefully consider the possibility of a Bochdalek hernia when evaluating patients with similar symptoms. This case study also illustrates the success of a minimally invasive surgical approach for repairing a Bochdalek hernia, with the use of laparoscopic techniques and using falciform ligament to support the diaphragm.

## Introduction

Hiatal hernia and Bochdalek hernia are two types of congenital diaphragmatic hernias. While hiatal hernia occurs commonly in adults and is usually acquired, Bochdalek hernia is a rare type of congenital diaphragmatic hernia that is usually diagnosed in childhood. Hiatal hernias occur when the stomach protrudes through the diaphragmatic esophageal hiatus into the thoracic cavity, while Bochdalek hernias occur when abdominal contents herniate through the posterolateral segment of the diaphragm [1].

Hiatal hernias can cause symptoms such as heartburn, regurgitation, and difficulty swallowing. On the other hand, Bochdalek hernias can lead to respiratory distress and gastrointestinal symptoms such as vomiting, abdominal pain, and feeding difficulties. Hiatal hernias are repaired surgically through various procedures, usually in concurrence with laparoscopic Nissen fundoplication and transoral incisionless fundoplication (TIF), whereas Bochdalek hernias require immediate surgical intervention due to the potential for incarceration, strangulation, and lung hypoplasia. Hiatal hernias are more common in adults and rarely lead to life-threatening complications, whereas Bochdalek hernias can be lethal if not promptly detected and treated [1].

Bochdalek hernias are often incidental findings on imaging studies or discovered in the workup of respiratory distress, whereas hiatal hernias are typically diagnosed through endoscopic evaluation. An adult Bochdalek hernia is rare and can present as a result of the iatrogenic weakness of the diaphragm due to major surgery. A recent paper by Hagiwara et al. [2] described a case of an adult patient who presented with respiratory distress and was diagnosed with an undiscovered Bochdalek hernia. The patient was an 86-year-old male who had presented with right-sided heart failure. He had a coronary artery bypass with gastroepiploic artery 20 years prior. Following the surgery, he developed recurrent pneumonia and was eventually diagnosed with a Bochdalek hernia.

Imaging studies, such as ultrasound and CT scans, can be used to diagnose Bochdalek hernias in both children and adults. But these diagnostic methods are not always effective, and clinical suspicion should also be considered in the differential diagnosis. A CT scan is the most definitive diagnostic tool, as it can show fat above the diaphragm with possible organ entrapment. A paper by Hamid et al. [3] discusses the importance of considering Bochdalek hernia in adult patients presenting with respiratory distress or gastrointestinal symptoms that cannot be explained by other underlying conditions.

A study by Katsaros et al. [4] emphasizes the importance of prompt surgical intervention in patients diagnosed with Bochdalek hernia to avoid potentially life-threatening complications. These hernias can escape detection in adults and become surgical emergencies when abdominal organs are strangled, leading to serious medical conditions such as intestinal obstruction or gastric volvulus. A surgical repair is the only effective treatment for Bochdalek hernia in adults, as it can prevent long-term complications and improve quality of life. In regards to the type of surgical approach, a laparoscopic approach may be favored by experienced surgeons due to its lesser invasiveness and faster recovery rates. A paper by Machado [5] suggests that a minimally invasive approach can be successful in treating Bochdalek hernias detected incidentally in imaging studies or during surgery in adults.

## Case presentation

Our case details a 64-year-old female patient who was diagnosed with chronic obstructive pulmonary disease, sleep apnea, hypothyroidism, and hypertension. She was first referred to a pulmonologist by her primary care physician due to symptoms of worsening oxygen concentration at rest. She was then put on nasal oxygen therapy, but her symptoms continued to worsen. The pulmonologist ordered a CT scan, which diagnosed her as having a large, 8 cm hiatal hernia.

She also suffered from symptoms of gastroesophageal reflux disease, including frequent heartburn, regurgitation of food, and difficulty swallowing. In view of these pre-existing conditions, she was scheduled to have a laparoscopic hiatal hernia repair along with a TIF by a surgeon. Upon meeting her surgeon, the patient complained of prolonged chest pain and worsening dyspepsia. Cardiac clearance was obtained and surgery was scheduled.

Prior to the surgery, informed and written consent was taken. Preoperative antibiotics and anesthesia were given intravenously. During the surgery, the surgeon discovered what was thought to be the hiatal hernia was a large Bochdalek hernia measuring approximately 8 cm that had gone undiagnosed for decades. The patient's stomach, small intestine, and a portion of the colon were completely herniated into the thoracic cavity, leading to strangulation of the bowel and a life-threatening emergency. The surgeon tried to continue the laparoscopic repair, but it proved to be difficult due to the size and severity of the hernia. The surgeon then tried performing the repair by using a biologic mesh, but this method also faced complications due to the distorted anatomy of the patient's chest. In this situation, the surgeon continued the laparoscopic procedure and attempted to use the falciform ligament on the liver to reinforce the diaphragmatic defect, since it was not possible to use the mesh due to adhesions.

**Figure 1:**
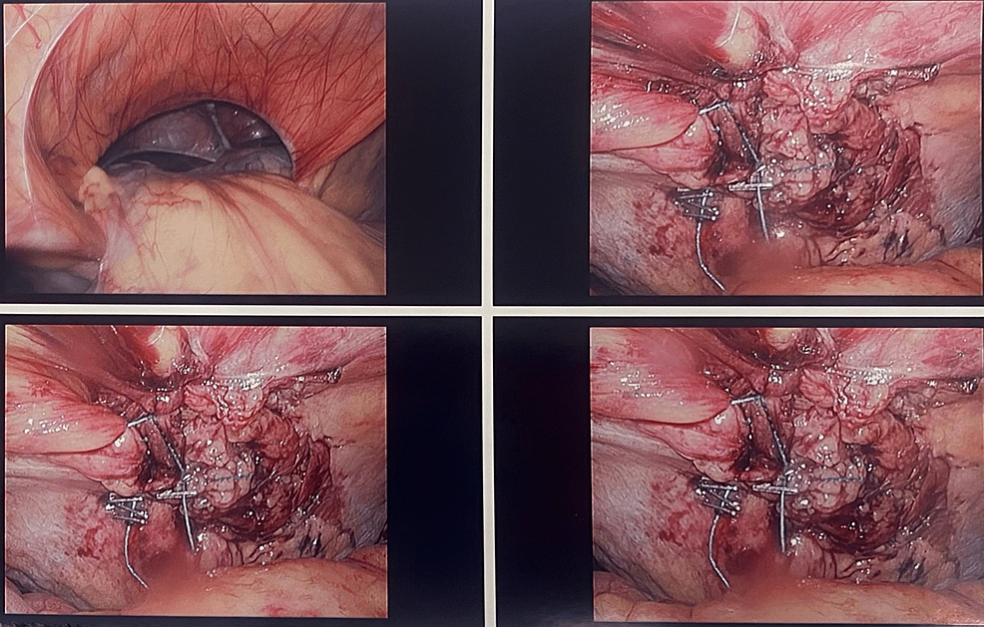
From top left, clockwise: contents (stomach, small intestines) being evacuated from the hernial cavity, buttress stitching of the hernia in stages.

The falciform ligament was carefully dissected and used as a patch to close the Bochdalek hernia defect. This method worked to close the defect due to the presence of a "buttressing effect" of the liver, preventing abdominal viscera from being displaced into the thoracic cavity. Once the Bochdalek hernia was repaired, the surgeon performed a flexible upper endoscopic procedure to check for the presence of any additional hernia or pathology in the patient's gastrointestinal tract. At this point, the hiatal hernia was not visible and no gastroesophageal bleeding was seen. The TIF procedure was considered to not be necessary in this case, and the surgeon opted to close the patient's surgical incisions instead. A summary of the steps of surgery is shown in Figure 1.

The patient was kept overnight in the hospital for observation and given antibiotics to prevent infection. No postoperative complications were seen, and the patient was discharged with appropriate pain management and instructions for follow-up care. This case highlights the importance of diagnosis of Bochdalek hernias as the patient had severe symptoms of gastroesophageal reflux disease, yet the underlying cause was mistaken for a hiatal hernia. In addition, since the Bochdalek hernia was not diagnosed earlier, the patient suffered respiratory and digestive complications due to the prolonged displacement of the large intestine into the thoracic cavity. During the one-month follow-up at the ambulatory center, the patient reported remarkable improvement in her dyspnea, which was grade 4 prior to surgery and was evaluated to have changed to stage 2 at the follow-up appointment.

This case details how the lack of specific diagnostic techniques for Bochdalek hernia in adults, along with its rarity and atypical presentation, can lead to diagnosis delay. Furthermore, this case also shows the importance of surgical expertise and adaptability in dealing with unexpected complications during surgical procedures.

## Discussion

Bochdalek hernias are rarely seen in adults and are most often misdiagnosed as other conditions such as hiatal hernias or gastroesophageal reflux disease. They present with similar symptoms, such as chest pain and respiratory or gastrointestinal symptoms. A paper by Gue et al. [6] emphasizes that Bochdalek hernias may present with a number of non-specific chronic conditions and that surveillance and long-term follow-up are required for most patients. Our case had a similar finding since the patient was known to be a long-standing sufferer of gastroesophageal reflux disease as well as chronic obstructive pulmonary disease, and it was not until the surgical intervention that her Bochdalek hernia was detected.

It is also important that Bochdalek hernias are treated promptly when discovered, either incidentally or otherwise. A case study by Testini et al. [7] detailed how the preferred method of treatment was a surgical approach, usually done because of an incidental finding during imaging or thoracoabdominal surgeries. A similar finding was reported in our case where the patient was operated upon immediately when the hernia was first diagnosed.

Bochdalek hernias also have been treated either with open surgery or a laparoscopic approach. This is further supported by Lau et al. [8], who report that a minimally invasive approach such as laparoscopy can be used in settings where no emergency bowel resection is warranted. Our case also has similar findings in that a laparoscopic approach worked well for the patient and no postoperative complications were seen.

Previous literature by Gale et al. [9] suggests that a CT scan is the most reliable diagnostic tool for Bochdalek hernias. The paper also talks about how Bochdalek hernias are easily distinguishable on CT scans. Our paper is dissimilar in this aspect as the patient's preoperative CT scan showed no evidence of a Bochdalek hernia, and it was only during the surgery that the hernia was discovered.

A brief summary of previous Bochdalek hernia cases, which have similar findings to ours, has been illustrated in Table 1 below. These cases are important in understanding the need for prompt diagnosis and permissible treatments when an adult presents with atypical symptoms.

**Table 1:** Summary of prior Bochdalek hernia cases

Ref. No.	Name of the case report	Brief case details	Conclusions
[6]	Bochdalek hernia in an adult female with intrathoracic left kidney and splenic flexure of the colon	Bochdalek hernia in an adult female with intra-thoracic left kidney and splenic flexure of the colon, who presented with non-specific abdominal symptoms.	Contrast-enhanced CT scan of the abdomen in the management of all cases with non-specific abdominal symptoms.
[7]	Obstructed Bochdalek hernia in an adult	Case of a woman who presented with an acutely obstructed posterolateral diaphragmatic hernia. The initial physical exam and radiological results could have led to an erroneous diagnosis of pneumothorax.	Emphasize the importance of having a high index of suspicion for this condition when cases with similar gastro-respiratory symptoms are encountered.
[8]	Laparoscopic repair of colonoscopy-induced adult Bochdalek hernia	Case of delayed Bochdalek hernia that may have been induced by the difficult insertion of a colonoscopy.	Not possible to have been a colonoscopy injury. Disorders should be recognized, examined, and treated appropriately to avoid complications.
[9]	Bochdalek hernia in a young adult successfully treated with a laparoscopic approach	An otherwise healthy patient without past medical history; after vigorous physical activity, a Bochdalek hernia was discovered and successfully treated.	Modern surgical techniques can improve patient recovery and short hospital stay with minimal morbidity or mortality.

Learning points

This case highlights the importance of diagnosis of Bochdalek hernias as the patient had severe symptoms of gastroesophageal reflux disease, yet the underlying cause was mistaken for a hiatal hernia. It also emphasizes the need for vigilance and follow-up in patients with non-specific chronic conditions where Bochdalek hernias may be undetected. In addition, since the Bochdalek hernia was not diagnosed earlier, the patient suffered respiratory and digestive complications due to the prolonged displacement of the large intestine into the thoracic cavity. This case details how the lack of specific diagnostic techniques for Bochdalek hernia in adults, along with its rarity and atypical presentation, can lead to diagnosis delay. Furthermore, this case also shows the importance of surgical expertise and adaptability in dealing with unexpected complications during surgical procedures. A minimally invasive method worked well in our patient since no bowel obstruction was seen. Future research should focus on identifying high-risk groups for Bochdalek hernias and establishing screening protocols to detect the condition early.

## Conclusions

Our case highlights the importance of accurate diagnosis of Bochdalek hernias in adults, and also the need for prompt treatment when incidental findings occur. A minimally invasive method worked well in our patient since no bowel obstruction was seen. Moreover, our case also emphasizes the need for vigilance and follow-up in patients with non-specific chronic conditions where Bochdalek hernias may be undetected. Future research should focus on identifying high-risk groups for Bochdalek hernias and establishing screening protocols to detect the condition early.
